# Leader Humility and Taking Charge: The Role of OBSE and Leader Prototypicality

**DOI:** 10.3389/fpsyg.2019.02515

**Published:** 2019-11-22

**Authors:** Wenwen Zhang, Wenxing Liu

**Affiliations:** ^1^School of Management, Huazhong University of Science and Technology, Wuhan, China; ^2^School of Business Administration, Zhongnan University of Economics and Law, Wuhan, China

**Keywords:** leader humility, taking charge, organizational-based self-esteem, leader prototypicality, self-concept-based theory

## Abstract

Taking charge refers to an extra role behavior that is change oriented and can bring constructive benefits to the organization. However, taking charge always involves risks and might incur potential costs for employees. Understanding how to encourage employees’ taking charge has become increasingly important for today’s organizations. Drawing on self-concept-based theory, we intend to explore when and why leader humility would inspire followers’ taking charge behavior in China. Employing a time-lagged research design with a sample of 190 supervisor-subordinate dyads, we found that the association between leader humility and taking charge is significantly and positively correlated, with organization-based self-esteem (OBSE) mediating the connection. We also found support for the moderating effect of leader prototypicality. Leader humility positively affected taking charge via followers’ OBSE, though only in cases of high leader prototypicality. Finally, we probed into the practical and theoretical implications of this research.

## Introduction

Over the last decade, organizations worldwide have witnessed a tremendous change in the business environment, especially under the impact of a global economic crisis. Scholars have documented that such economic change would have a significant impact on organizations and workers around the world (Burns et al., 2012; Giorgi et al., 2015; Mucci et al., 2016). For employees, they might face the reality of job insecurity, job uncertainty, and even unemployment, which would have a significant impact on the attitudes, cognitions, and behaviors of employees (Mucci et al., 2016). For organizations, in order to maintain competitiveness in the context of a global economic crisis, organizations are becoming more and more reliant on the proactive behaviors of their members to develop further (Grant et al., 2009). Taking charge, as one form of proactive behavior, has stimulated plenty of research interest over the past decades (e.g., Burnett et al., 2015; Li et al., 2016). In line with Morrison and Phelps (1999), taking charge is the “employees’ voluntary behavior that usually change[s] work processes, policies and routines and aims at challenging current conditions.” On one hand, taking charge can bring about constructive changes in the workplace and benefit long-term organizational adaptability. On the other hand, taking charge involves potential political and image risks, and it might incur costs for employees through a violation of group expectation. Therefore, when deciding whether to engage in taking charge, employees will weigh anticipated costs against anticipated benefits (Morrison and Phelps, 1999). Thus, taking charge cannot emerge naturally, it needs to be activated by external stimuli.

Recently, research on taking charge has primarily focused on exploring its antecedents and underlying mechanism from personal and situational factors (Parker et al., 2006; Burnett et al., 2015; Li et al., 2016). Leadership, which is argued to be an important contextual factor, would play an essential role in employee behavior. Although recent research has shown that various leadership approaches, for instance, transformational leadership (Li et al., 2013), self-sacrificial leadership (Li et al., 2016), and empowerment leadership (Qian et al., 2018), would be beneficial to followers’ taking charge, much less is known about how leader humility would elicit employees’ taking charge. Leader humility, in line with Owens et al. (2013), is an interpersonal trait in which leaders are more likely to think objectively of themselves, have a tendency to appreciate the excellence and contributions of others, and are inclined to be open to new opinions and suggestions. In comparison with the aforementioned leadership modes, leader humility literally implies leading from the bottom to the top (Owens et al., 2013). Humble leaders might provide more emotional resources to employees through building a close relationship with them, which increases the likelihood for employees to bring constructive benefits to the organization. Furthermore, humble leaders are more likely to think objectively of themselves and always ask employees to remedy or compensate for their weaknesses (Owens and Hekman, 2012), which might make employees feel obligated to bring about constructive change.

Moreover, studies on leader humility have almost adopted a leader-centered perspective, revealing the effect of leaders’ actions on the attitudes of their employees and their consequent evaluation of their leaders. For example, scholars have documented the positive influence of leader humility on team performance (Chiu et al., 2016), employees’ relational energy (Wang et al., 2018), and followers’ harmonious passion (Diao et al., 2019). However, relatively little attention has been given to the exploration of how leader humility affects the self-assessment of employees (Bono and Judge, 2003). Specifically, drawing on self-concept-based theory, the current study proposes a model to investigate how leader humility affects taking charge via organization-based self-esteem (OBSE) (Pierce et al., 1989). OBSE is defined as the extent to which organizational members recognize themselves as competent, important, and valuable (Pierce et al., 1993). We argue that leader humility would promote OBSE in followers and their subsequent taking charge.

**FIGURE 1:**
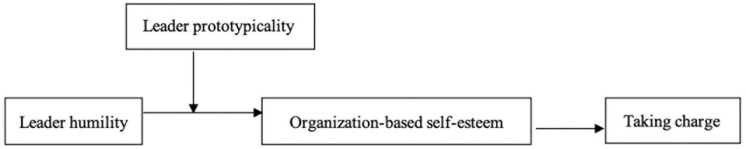
Theoretical model.

Finally, we also seek to explore under what conditions the effect of leader humility on taking charge is more evidenced. We attempt to address whether leader prototypicality (i.e., “the extent to which leaders is on behalf of the group identity”) moderates the relationship between leader humility and taking charge (Van Knippenberg and Hogg, 2003). Prior research has reported that the more the group leaders are prototypical the more they are recognized as more effective (De Cremer et al., 2010). We therefore hypothesize that, when they are perceived as being more prototypical, humble leaders are more effective in facilitating OBSE in followers and consequently in taking charge. The overall proposal of this study is presented in Figure 1.

Our investigation intends to complement prior research in three ways. Foremost, by probing into the influence of leader humility on followers’ taking charge, our study complements the research on the effect of leadership approaches on subordinate proactive behavior. It seems that the current study is the first attempt to address the link between leader humility and taking charge. Second, in view of self-concept-based theory, we can provide a new perspective to explore the psychological mechanism through which “bottom–up” leader humility impacts taking charge. Finally, to acquire a better understanding of leader humility, scholars have called for more studies to investigate the boundary conditions of leader humility (Owens and Hekman, 2012). The present study identifies the moderating role of leader prototypicality, which represents a new boundary condition for the relationship between leader humility and taking charge.

## Theoretical Background and Hypotheses

### Self-Concept-Based Theory

The self-concept-based theory originated from Bandura’s (1989) social cognitive theory and sociological literature on the self-concept (Shamir et al., 1993). By investigating the motivational mechanism between leaders and their followers, Shamir et al. (1993) employed self-concept-based theory to advance transformational leadership research. In line with self-concept-based theory, leaders can influence their followers’ motivation through three ways: improving their confidence, promoting their social identity with the organization, and integrating work values with their own values (Bono and Judge, 2003). Shamir et al. (1993) asserted that leaders’ behavior plays an important role in followers’ self-concepts, identities, and values, which then all significantly influence followers’ reactions.

In the current study, in line with self-concept-based theory, we aim to explain how leader humility activates followers’ self-concept-related motivations (OBSE) and their subsequent behavior. OBSE refers to the employees’ sense of being valuable and competent as organizational members, and it thus has strong implications for work-related motivation, attitudes, and behaviors. Moreover, we employ leader prototypicality to uncover the boundary condition of the effectiveness of leader humility.

### Taking Charge

In line with Morrison and Phelps (1999), taking charge is the “employees’ voluntary behavior that usually change[s] work processes, policies and routines and aims at challenging current conditions.” As one type of proactive behavior, taking charge has some distinction from OCB and other related constructs such as voice, innovation, issue selling, principled dissent, whistle blowing, task revision, and personal initiative (Morrison and Phelps, 1999). Two judgments will affect the decision to take charge: evaluating the likelihood of success and the costs of the behavior. Past research has demonstrated that not only individual characteristics but also contextual factors would play an important role in taking charge (Parker et al., 2006; Burnett et al., 2015; Li et al., 2016). Employees who are self-efficacious and who have a strong sense of duty would be more inclined to take charge (Morrison and Phelps, 1999). Moon et al. (2008) found that, in contrast to achievement striving (which focuses on personal interest), duty (which focuses on others’ interest) was positively associated with taking charge. Researchers have also shown that contextual influences such as group norms that support change (Morrison and Phelps, 1999), procedural justice (Moon et al., 2008), transformational leadership (Li et al., 2013), and self-sacrificial leadership (Li et al., 2016) were positively associated with taking charge. It appears that taking charge is much more likely to occur when employees believe that change can and will be enacted. Based on self-concept-based theory, this paper tries to investigate the relationship between leader humility and employees’ taking charge from a follower-centric perspective, which centers around how employees’ self-assessment is shaped by leaders’ behavior (Pierce and Gardner, 2004) and thus their reactions.

### Leader Humility and Subordinate Taking Charge

According to Owens et al.’s (2013) definition, leader humility refers to “an interpersonal trait in which leaders are more possibly to objectively think of themselves, have a tendency to appreciate others’ excellences and contributions, and are inclined to be open to new opinions and suggestions” (Owens et al., 2013). Humble leaders are more willing to objectively evaluate themselves, display an appreciation of followers’ strengths and contributions, and have a high interest in learning from others (Owens and Hekman, 2012). Prior research has shown that leader humility has a positive impact on motivating employees’ voice behavior (Lin et al., 2017), which is a form of proactive behavior. However, according to our knowledge, research has yet to examine the relationship between leader humility and other proactive behavior (for instance, taking charge). Researchers have distinguished taking charge from voice behavior in that taking charge emphasizes bringing constructive changes to organizations (Morrison and Phelps, 1999), whereas the objective of voice is not to bring about organizational improvement but to enhance personal satisfaction (Withey and Cooper, 1989).

Moreover, engaging in taking charge also involves certain risks (Morrison and Phelps, 1999; McAllister et al., 2007). As a form of challenging proactive behavior, taking charge focuses on changing the current situation such that it may provoke conflicts or cause damage to the existing relationships, for example, to the extent that leaders may not like employees to go beyond the call of duty. Such discretionary behavior might incur great risks for employees. Therefore, we posit that leader humility would motivate employees’ taking charge in two ways. First, humble leaders are more willing to appreciate followers’ competences and contributions, which make employees feel that their work is valued and recognized by the organization. This would increase the likelihood of employees putting more effort in to better serve the organization. Therefore, employees are motivated to initiate proactive behavior to bring constructive benefits to the organization. Second, humble leaders are more likely to objectively think of themselves and always ask employees to remedy or compensate for their weaknesses (Owens and Hekman, 2012). Thus, employees might feel obligated to bring about constructive change. When employees felt responsibility regarding change, they will believe that taking charge is more likely to succeed. Research has indicated that followers are more inclined to taking charge when there is a high likelihood of success.

**Hypothesis 1:** Leader humility is positively related to followers’ taking charge.

### The Mediating Role of OBSE

Organization-based self-esteem explicitly refers to the extent to which organizational members recognize themselves as competent, important, and valuable (Pierce et al., 1993). We chose OBSE rather than a general form of self-esteem because the former is more suitable for organizational settings (Pierce et al., 1993). Empirically, scholars have demonstrated that there is a relationship between OBSE and intrinsic motivation, organizational citizenship, feedback-seeking behavior, performance, and organizational commitment (Pierce and Gardner, 2004).

Prior research has shown that influential and vital others are major sources that OBSE comes from Baumeister (1999). In the organizational settings, leaders are always recognized as the influential and vital others. Specifically, in the current study, humble leaders are presumed to play an essential role in motivating OBSE in subordinates. When humble leaders think that a subordinate is capable, competent, and need satisfying and communicate these perceptions through words and behaviors, it will make subordinates have a similar self-evaluation (Pierce and Gardner, 2004). Thus, the OBSE of subordinates is shaped partially by the messages sent by humble leaders. When subordinates combine and incorporate such information into their own minds, they would have the self-concept as those evidenced in leaders’ words and behaviors (Shamir et al., 1993; Pierce and Gardner, 2004). Furthermore, the positive self-concept would have significant impact on subsequent behaviors.

#### Leader Humility and Organization-Based Self-Esteem

In this article, we propose that leader humility would enhance followers’ OBSE. In line with Owens et al. (2013), leader humility is an interpersonal trait that is manifested in (1) admitting personal limitations and shortcomings; (2) highlighting follower excellence and contributions; and (3) modeling teachability. These three interconnected components of leader humility capture the core elements of this bottom–up leadership approach. In addition, due to the fact that leader humility legitimizes follower growth and development, humble leaders would have an efficient impact on their employees’ perceptions and actions (Owens et al., 2013). Thus, we argue that leader humility might motivate followers’ OBSE in three ways.

First, humble leaders are more likely to acknowledge personal limits, faults, and mistakes. By admitting to personal weakness, humble leaders can objectively evaluate themselves, even to the extent that humble leaders might ask followers to help them remedy or compensate for a weakness (Owens and Hekman, 2012). We argue that this aspect of leader humility would increase employees’ OBSE. Further, employees tend to believe that leaders who are willing to admit personal vulnerabilities are more likely to value their suggestions and behaviors. As a result, employees might form a sense of competence and thus heightened OBSE in their organizations (Pierce and Gardner, 2004).

Second, through appreciating followers’ strengths and contributions, humble leaders communicate to employees that their job contributions are valued and recognized (Owens and Hekman, 2012), thus leading to an elevated sense of OBSE. Further, humble leaders are more open to uncertainty and value employees’ endeavors in changing present work conditions. Not only do these behaviors afford employees with strong mental resources to consistently enhance personal capability, but they also make employees aware of their contribution to the organization. As a result, employees would incorporate humble leaders’ perceptions of their capacities into self-concept, thus further increasing employees’ sense of competence, self-image, and self-evaluation (Pierce and Gardner, 2004).

Finally, humble leaders are open-minded and have a habit of listening to new ideas and information before speaking (Owens and Hekman, 2012). Rather than merely telling followers how to do things, humble leaders always model follower tasks and seek feedback from the follower. Through modeling, humble leaders also initiate role reversals with followers, which means putting followers in the leader role and leaders in the follower role. This makes employees feel that they can truly do something, and their suggestions and behaviors would be taken into serious consideration by humble leaders. Therefore, employees would feel valuable and competent in their organizations, thus leading to high self-evaluation and an elevated sense of OBSE (Pierce and Gardner, 2004). Thus, by admitting personal limitations and mistakes, being teachable, and by recognizing employees’ strengths and contributions, humble leaders can influence employees’ OBSE.

#### Organization-Based Self-Esteem and Taking Charge

Organization-based self-esteem, as an important self-evaluation construct, has been argued to have implications on subsequent motivation, attitudes, and work-related behaviors. Prior research has shown that OBSE can influence intrinsic motivation, organizational citizenship, feedback seeking behavior, performance, and organizational commitment (Pierce and Gardner, 2004).

As mentioned above, we have theorized how leader humility would enhance followers’ OBSE. In the following we attempt to postulate how increased OBSE would give rise to individuals taking charge. First, with high levels of OBSE, employees recognize themselves as competent, important group members (Pierce and Gardner, 2004). In other words, employees with an elevated sense of OBSE might have a sense of control and be much more confident in the likelihood of success. Furthermore, employees with a greater awareness of OBSE are more apt to overcome the psychological barriers of, and more inclined to perceive the possible advantages of engaging in taking charge. Thus, employees with a positive self-concept are motivated to improve their work environment and proactively seek opportunities to make effective changes to works methods, policies and procedures.

Second, in line with self-concept-based theory (Shamir et al., 1993), employees with high levels of OBSE have a strong desire to maintain self-consistency. Employees with elevated OBSE tend to be confident in their capacities and have gained a feeling of gratification during their work experiences. They behave in ways that maintain their level of OBSE. Thus, in striving for self-consistency (Korman, 1970), employees with high levels of OBSE are inspired to accomplish work to the best of their abilities and are more likely to throw themselves into behaviors that are beneficial to the organization (e.g., taking charge). Taken together, we hypothesize the following:

**Hypothesis 2:** Followers’ OBSE mediates the positive relationship between leader humility and followers’ taking charge.

### Leader Prototypicality as the Moderator

Leader prototypicality refers to the degree to which leaders represent the group identity (Van Knippenberg and Hogg, 2003). According to social identity theory of leadership, apart from proving guidelines to employees in the group, leaders are also part of the group (Van Knippenberg and Hogg, 2003; Van Knippenberg, 2011). When leaders are considered to have high levels of leader prototypicality (i.e., representative of the group identity), they might be more efficient in motivating and inspiring followers. Furthermore, followers’ perceptions of their own status can also be positively shaped by leader prototypicality (Van Dijke and De Cremer, 2008).

In essence, as part of the group, prototypical leaders can so optimally embody the group’s identity so that their behaviors and decisions might signal the group opinion. Compared with leaders who are perceived to have low levels of leader prototypicality, leaders with high levels of leader prototypicality can significantly help employees eliminate the uncertainty of their own self-concept. Therefore, being representative of the group’s identity, humble leaders’ behavior, such as reflecting on themselves, highlighting followers’ strengths and contributions, and being teachable, further facilitates subordinates’ OBSE. Therefore, we argue that leader prototypicality would function as a moderator between the relationship between leader humility and employees’ OBSE.

**Hypothesis 3:** Leader prototypicality moderates the positive relationship between leader humility and OBSE so that the relationship is stronger for leaders with high levels of prototypicality (in contrast to leaders with low levels of prototypicality).

Moreover, leader prototypicality would also have impact on the indirect relationship between leader humility and taking charge. Specifically, leader prototypicality would reinforce the impact of leader humility on taking charge via OBSE. When humble leaders are perceived to have high levels of leader prototypicality, it might increase the subordinates’ willingness to engage in taking charge via OBSE.

**Hypothesis 4:** Leader prototypicality moderates the positive impact of leader humility on taking charge via followers’ OBSE so that the relationship is stronger for leaders with high levels of prototypicality (in contrast to leaders with low levels of prototypicality).

## Materials and Methods

### Sample and Procedure

For the sake of overcoming the question regarding common method bias, the current study adopted a two-wave supervisor-subordinate dyads questionnaire research design. With the admission of a large manufacturing company located in Central China, we were capable of collecting data from both the production and R&D personnel. Prior to proceeding with the survey, the second author of this study carries out a briefing session. He emphasized that each employee’s participation was valued and that participation in the survey was voluntary. He also guaranteed that all the information collected would be used for academic research exclusively. Data were collected in two sessions with a 2-week interval. Each participant was required to put the questionnaires in an enclosed envelop and give it back directly to the coordinators right after completion. We designed and distributed the leadership questionnaire and the subordinate questionnaire. In the first wave (Time 1), subordinates were required to offer their personal information and evaluate leader humility, OBSE, and leader prototypicality. In the second wave (Time 2), supervisors were asked to assess subordinates’ taking charge. We had to write the names of the specific subordinates on the supervisor questionnaire first, and we then asked the supervisor to erase these names after completion. Supervisors also need to provide their own background information in the questionnaire.

At time 1, we distributed a total of 260 questionnaires and we received all the questionnaires back. At time 2, 200 supervisor questionnaires were returned, with a response rate of approximately 77%. After deleting invalid cases and unmatched data, 190 supervisor-subordinate dyads remained. The average age of employees is 33 years old (*SD* = 7.95). In terms of education, more than half of people have junior college or bachelor’s degrees (50.5%). The average organizational tenure of employees is 7.68 years (*SD* = 7.05), and the mean team tenure is 5.26 years (*SD* = 5.31).

### Ethics Statement

The Huazhong University of Science and Technology’s Ethics Committee approved our research design and declared that this study did not violate any legal regulations or common ethical guidelines. Before the conduction of the survey, we also got approval from the firm and all the employees. We emphasized that each employee’s participation was valued and that all participation was voluntary. We also guaranteed that all the information collected would be used for academic research exclusively.

### Measures

In line with traditional practices, we employ the conventional method of back translation to translate the English language questionnaires (Brislin, 1986). Two bilingual Chinese scholars independently conducted this work.

#### Leader Humility

We adopted Owens et al.’s (2013) nine-item scale to measure leader humility. Subordinates were asked to rate the items on a six-point Likert scale (1 = totally disagree, 6 = totally agree). Sample items include “My leader shows appreciation for the unique contributions of others,” and “My leader is open to the advice of others.” The Cronbach’s alpha coefficient for this measure was 0.96.

#### Organization-Based Self-Esteem

We measured OBSE using the 10-item scale developed by Pierce et al. (1989). Subordinates were asked to rate on a six-point Likert scale (1 = totally disagree, 6 = totally agree). Sample items include “I am helpful around here.” The Cronbach’s alpha coefficient for this measure was 0.84.

#### Taking Charge

To measure followers’ taking charge, we employed the 10-item scale developed by Morrison and Phelps (1999). Leaders were required to rate on a six-point Likert scale (1 = totally disagree, 6 = totally agree). Sample items include “This person often tries to change how his or her job is executed in order be more effective” and “This person often try to adopt improved procedures for doing his or her job.” The Cronbach’s alpha coefficient for this measure was 0.91.

#### Leader Prototypicality

We adopted the four-item scale used by Steffens et al. (2014) to assess leaders’ prototypicality. Followers were required to rate on a six-point Likert scale (1 = totally disagree, 6 = totally agree). Sample items include “My supervisor embodies what this team stands for.” The Cronbach’s alpha coefficient for this measure was 0.95.

#### Control Variables

Consistent with past research (Pierce and Gardner, 2004; Owens et al., 2013), we controlled for multiple factors that might impact our estimation results, including the subordinates’ gender, age, education, organization tenure, and team tenure.

### Measurement Model

In order to verify the validity of these constructs in the present study, we conducted a CFA using Mplus 7.4. As indicated in Table 1, the CFA showed that the four-factor model fitted to the data much better (χ^2^ = 212.2, df = 59, CFI = 0.93, TLI = 0.91, RMSEA = 0.11) than the six three-factor models in which any two of the four factors were combined. In contrast to the model with two factors (where leader humility and leader prototypicality were combined, OBSE and taking charge were combined; χ^2^ = 1195.24, df = 64, CFI = 0.49 TLI = 0.37, RMSEA = 0.30 Δχ^2^ = 983.04) and the model with one factor (χ^2^ = 1425.83, df = 65, CFI = 0.38, TLI = 0.26, RMSEA = 0.233, Δχ^2^ = 1213.63), the four-factor model is more appropriate. The above results revealed that the model we proposed has the best validity.

**TABLE 1:** Confirmatory factor analyses.

**Model**	**χ^2^**	**df**	**χ^2^/df**	**CFI**	**TLI**	**RMSEA**
Four-factor model (LH; OBSE; TC; LP)	212.20	59	3.60	0.93	0.91	0.12
Three-factor model (LH + OBSE; TC; LP)	446.89	62	7.21	0.83	0.78	0.18
Three-factor model (LH + TC; OBSE; LP)	762.98	62	12.31	0.68	0.60	0.25
Three-factor model (LH + LP; OBSE; TC; LP)	751.32	62	12.12	0.69	0.61	0.24
Three-factor model (LH; OBSE + TC; LP)	555.14	62	8.96	0.78	0.72	0.21
Three-factor model (LH; OBSE + LP; TC)	617.81	62	9.97	0.75	0.69	0.22
Three-factor model (LH; OBSE; TC + LP)	917.59	62	14.80	0.62	0.52	0.27
Two-factor model (LH + LP; OBSE + TC)	1039.54	64	16.24	0.56	0.47	0.29
One-factor model (LH + OBSE + TC + LP)	1425.83	65	21.94	0.39	0.27	0.34

**N* = 190. LH, leader humility; OBSE, organization-based self-esteem; TC, taking charge; LP, leader prototypicality; CFI, Comparative Fit Index; TLI, Tucker-Lewis Index; RMSEA, root-mean squared error of approximation.*

## Results

### Descriptive Statistics

Means, standard deviations, and correlations among the variables of interest are presented in Table 2. Leader humility was significantly associated with followers taking charge (*r* = 0.28, *p* < 0.01) and OBSE (*r* = 0.58, *p* < 0.01). Organization-based self-esteem was significantly related to taking charge behavior (*r* = 0.34, *p* < 0.01).

**TABLE 2:** Means standard deviations and interrelations of variables.

**Variable**	***M***	***SD***	**1**	**2**	**3**	**4**	**5**	**6**	**7**	**8**	**9**
(1) Gender	0.43	0.50									
(2) Age	32.95	7.95	–0.02								
(3) Education	0.51	0.50	–0.21^∗∗^	–0.49^∗∗^							
(4) Org. tenure	7.68	7.05	0.01	0.80^∗∗^	–0.42^∗∗^						
(5) Group tenure	5.26	5.31	0.00	0.67^∗∗^	–0.34^∗∗^	0.73^∗∗^					
(6) Leader humility	4.96	1.04	–0.08	0.01	0.21^∗∗^	0.04	0.03	(0.96)			
(7) 0BSE	4.03	0.91	−0.18^∗^	0.01	0.12	0.02	0.10	0.58^∗∗^	(0.84)		
(8) Taking charge	4.51	0.94	–0.03	0.18^∗^	0.05	0.22^∗∗^	0.20^∗∗^	0.28^∗∗^	0.34^∗∗^	(0.91)	
(9) Leader prototypicality	2.35	1.02	0.13	0.17	–0.21	0.13	0.11	–0.35^∗∗^	–0.19^∗∗^	–0.22^∗∗^	(0.95)

**N* = 190, Reliabilities for the scales are in parentheses and presented along the diagonal. ^∗∗∗^*p* < 0.001; ^∗∗^*p* < 0.01; ^∗^*p* < 0.05.*

### Hypotheses Testing

We tested hypotheses 1 through a stepwise regression with SPSS22.0. Results of this analysis are presented in Table 3. After we controlled for the impact of subordinates’ gender, age, education, organizational tenure, and group tenure, results revealed that the effects of leader humility on follower taking charge was β = 0.23 (*p* < 0.001) (Table 3 Model 2). Thus, the results confirmed hypotheses 1.

**TABLE 3:** Results of stepwise regression.

	**Taking charge**		**OBSE**		
		
**Variable**	**Model 1**	**Model 2**	**Model 3**	**Model 4**	**Model 5**
Intercept	4.58^∗∗∗^ (0.07)	4.56^∗∗∗^ (0.07)	4.06^∗∗∗^ (0.07)	4.01^∗∗∗^ (0.06)	4.05^∗∗∗^ (0.06)
Gender	0.01 (0.07)	0.02 (0.07)	−0.15^∗^ (0.07)	−0.13^∗^ (0.06)	−0.12^∗^ (0.05)
Age	0.12 (0.13)	0.10 (0.13)	0.03 (0.13)	−0.00 (0.10)	−0.01 (0.10)
Education	0.75^∗^ (0.33)	0.49 (0.33)	0.42 (0.32)	−0.18 (0.27)	−0.19^∗^ (0.27)
Org. tenure	0.13 (0.12)	0.11 (0.12)	−0.11 (0.12)	−0.16 (0.10)	−0.14 (0.09)
Group tenure	0.09 (0.10)	0.09 (0.10)	0.18 (0.10)	0.18^∗^ (0.08)	0.16^∗^ (0.08)
Leader humility		0.23^∗∗∗^ (0.07)		0.52^∗∗∗^ (0.05)	0.46^∗∗∗^ (0.06)
Leader prototypicality					0.04 (0.06)
LH^∗^LP					0.12^∗∗^ (0.05)
*R*^2^	0.08	0.13	0.06	0.38	0.40
AR^2^	0.08^∗^	0.05^∗∗^	0.06^∗^	0.32^∗∗∗^	0.03^∗^

*^∗∗∗^*p* < 0.001; ^∗∗^*p* < 0.01; ^∗^*p* < 0.05.*

When it comes to examine the indirect effect of leader humility on subordinates taking charge via OBSE (hypotheses 2), we employed the bootstrapping method introduced by Preacher and Hayes (2004). With 5,000 bootstrapping tests, the data analysis showed that the indirect effect of leader humility on taking charge via OBSE was significant (β = 0.181, 95% confidence interval = [0.092,0.291]). Thus, the results support hypothesis 2.

**FIGURE 2:**
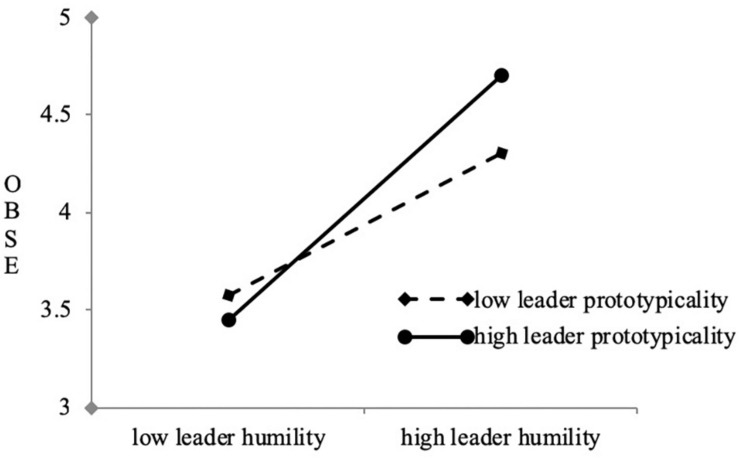
The moderating effect of leader prototypicality on leaderhumility and OBSE.

In order to confirm the moderation effect of leader prototypicality on the link between leader humility and OBSE (hypotheses 3), we performed stepwise regression analysis. We found that leader prototypicality significantly moderated the connection between leader humility and OBSE (β = 0.12, *p* < 0.01) (as evidenced in Table 3 Model 5). Next, we performed a simple slope analysis and portrayed the interaction effect in Figure 2. Results revealed that when leaders are perceived to have high levels of leader prototypicality, leader humility had a positive effect on OBSE.

Then, in line with the method introduced by Preacher and Hayes (2004), we performed an analysis to verify the conditional indirect effect (Hypotheses 4). The results were presented in Table 4. With 5,000 bootstrapping tests, we found that the conditional direct effect is 0.085 (95% confidence interval = [0.032,0.190]). Thus, hypotheses 4 was supported.

**TABLE 4:** Conditional indirect effect at specific values of leader prototypicality.

**Moderator**	**Level**	**Stage 1**		**Indirect effect**	
			
		**Estimate**	**95% CI**	**Estimate**	**95% CI**
Leader	High	0.645	[0.497, 0.785]	0.196	[0.091, 0.323]
	Low	0.367	[0.171, 0.560]	0.112	[0.044, 0.225]
	Diff.	0.278	[0.069, 0.378]	0.085	[0.032, 0.190]

*95% bias-corrected bootstrap confidence intervals are derived are based on 5,000 bootstrap samples.*

## Discussion

Through adopting the above theoretical model, we were able to acquire a better understanding of the underlying mechanism and boundary conditions about how leader humility stimulated subordinates taking charge. Data from a large manufacturing firm located in Central China help us confirm the hypotheses. The findings demonstrated that humble leaders positively influence employees’ taking charge, and OBSE acted as a moderator for this link. Furthermore, leader prototypicality moderated both the relationship between leader humility and OBSE and the indirect effect of leader humility on taking charge via OBSE. When leader prototypicality was perceived to be high, the positive relationships between leader humility and OBSE and the indirect effect of leader humility on subordinates taking charge via OBSE were stronger. The next part of this paper will probe into the theoretical and practical significance.

### Theoretical Implications

Through demonstrating the mediated effect of OBSE in the link between leader humility and followers’ taking charge, our research offers up some academic contributions to leader humility and taking charge studies. First, our research has further extended the research on proactive behavior. Previous studies have revealed that multiple leadership approaches would influence employees’ proactive behavior, including transformational leadership and participative leadership (Chia and Sharon, 2013). By exploring the impact of leader humility on followers’ taking charge, our research indicated the significant role of another unique and effective leadership approach—leader humility—in facilitating proactive employee outcomes.

Meanwhile, by adopting a self-concept-based theory as a theoretical foundation, we have employed a follower-centric approach to understand the potential impact of leader humility on taking charge. Our findings suggest that leader humility positively influenced followers’ taking charge through increasing followers’ OBSE. Such a finding supports the underlying assumption of self-concept-based theory that the self-assessment of follower function as a vital part at illustrating the influence of leadership on employees’ behavior (Shamir et al., 1993). In addition, the findings from the present study offer important theoretical insight about why employees working under humble leaders are more inclined to exhibit taking charge. This also help us gain more insight into the mechanism through which leader humility impacts employees. Moreover, past research regarding leader humility has primarily centered upon the influence of leader humility on work engagement and job satisfaction (Owens et al., 2013), team performance (Chiu et al., 2016), and firm performance (Ou et al., 2014). However, as organizational environments get more complex and unpredictable, organizations are becoming more and more reliant on employees’ proactive behaviors to develop and prosper (Grant, 2000; Grant et al., 2009). The positive influence of leader humility on followers’ taking charge revealed in the current study confirms our expectation that leader humility can facilitate the progress of taking charge. This result extends the range of behavioral outcomes stimulated by leader humility. Thus, our research findings extend the theoretical implications of leader humility.

Finally, our research also sheds light on the conditions in which leader humility is more likely to activate taking charge. Though prior research has already exhibited the positive effect of leader humility, they have given relatively little attention to the boundary conditions of leader humility (Owens and Hekman, 2012; Owens et al., 2015). Drawing from social identity analysis of leadership, we found that when humble leaders were recognized as having high levels of leader prototypicality, they would more effectively influence followers’ OBSE and the subsequent taking charge.

### Practical Implications

The current study provides insight into the essential role of humble leaders in motivating followers’ taking charge. Moreover, our research reveals that leader humility would impact taking charge through influencing subordinates’ self-evaluation. Given the recognized importance of taking charge in organizations, it seems necessary for organizations to pay increased attention to setting up effective procedures to select managers with humility. Furthermore, to motivate employees to engage in taking charge, humble leaders might publicly praise followers’ excellences and contributions and exhibit an attitude of openness to followers’ suggestions and feedback. These behaviors would have a positive impact on employees’ self-evaluation, thus making employees believe that they are competent, important, and valuable organizational members.

This study also reveals that the impact of leader humility on taking charge is heightened when leaders are perceived to have high levels of leader prototypicality. Specifically, when humble leaders are seen as more representative of the group identity, they would be more effective in motivating employees to engage in taking charge. Thus, when it comes to encouraging employees to bring more constructive benefits to the organization, humble leaders should pay more attention to the extent to which they represent the group’s identity and values.

### Limitations and Future Directions

Apart from these contributions of the current study, we admit that there also exist several limitations. First and foremost, we cannot confirm the causal links between leader humility and taking charge because of the cross-section properties of the data. Thus, we urge that scholars use longitudinal data or experimental studies to replicate the present study.

Second, we also need to urge caution regarding same-source bias. Although we have utilized a multi-source and time-lagged design in the data collection, we still cannot preclude the potential threat of same-source bias. Moreover, the antecedent (leader humility) and mediator (OBSE) were reported by subordinates at the same time so that the relationships among the variables might be inflated (Crampton and Wagner, 1994). Future research might pay more attention to this critical issue and invest more efforts in the section of research design.

Third, we only use data collected from a Chinese company to test our model so that the generalizability of the research findings might be a crucial issue. Due to the fact that the sample is limited to a specific economic context, it is possible that some of the findings cannot be applied to different settings. Therefore, we encourage future researchers to adopt samples from different countries to confirm the generalizability of the findings. Moreover, as a matter of fact, there exist many cultural influences, for example, power distance, that could impact the extent to which leader humility might stimulate followers’ taking charge. In future, we would therefore also suggest that researchers consider the impact of cultural factors.

Fourth, the dependent variable (i.e., taking charge) was reported by supervisors. To our knowledge, a growing number of researchers have employed leader’s evaluation as a means to assess follower’s behavior. But we also observe that several influences, such as the supervisors’ personal relationships with followers, may impact the ratings. For instance, having a low-quality leader-member exchange relationship with a leader might render the leader to make a biased assessment of followers’ taking charge. Therefore, future research could combine peer evaluation with the current method as a way to provide more robust findings.

## Conclusion

Drawing on a self-concept-based theory, we were able to explore the psychological mechanism and boundary conditions of leader humility. We demonstrated that leader humility leads to elevated perceptions of OBSE, which in turn causes a rise in taking charge, particularly when leaders are perceived to have high levels of leader prototypicality. In doing so, we provide initial support on why and when leader humility would incentivize followers’ taking charge. In the meantime, this study establishes a foundation for future research on understanding how leader humility can foster proactive action in organizations.

## Data Availability Statement

The datasets generated for this study are available on request to the corresponding author.

## Ethics Statement

Ethical review and approval was not required for the study on human participants in accordance with the local legislation and institutional requirements. The patients/participants provided their written informed consent to participate in this study.

## Author Contributions

WZ designed and wrote the manuscript. WL designed the study.

## Conflict of Interest

The authors declare that the research was conducted in the absence of any commercial or financial relationships that could be construed as a potential conflict of interest.
